# lncRNA MALAT1, HOTTIP and PVT1 as predictors for predicting the efficacy of GEM based chemotherapy in first-line treatment of pancreatic cancer patients

**DOI:** 10.18632/oncotarget.19345

**Published:** 2017-07-18

**Authors:** Cui-Juan Wang, Sheng-Bin Shi, Jing Tian, Jun Xu, Zuo-Xing Niu

**Affiliations:** ^1^ Shandong University of Traditional Chinese Medicine, Jinan, P.R. China; ^2^ Department of Medical Oncology, Shandong Cancer Hospital Affiliated to Shandong University, Jinan, P.R. China; ^3^ Central Laboratory, Shandong Academy of Occupational Health Medicine, Jinan, P.R. China

**Keywords:** advanced pancreatic cancer, GEM based chemotherapy, first-line therapy, lncRNA MALAT1, HOTTIP and PVT1

## Abstract

This study evaluated the lnc-RNAs as biomarker to predict efficacy of gemcitabine (GEM) based chemotherapy as the first-line treatment for locally advanced or advanced pancreatic cancer patients. We selected 62 patients with GEM based chemotherapy and divided two groups according to the PFS. We found that the expression of MALAT1, HOTTIP, and PVT1 in serum had a significant difference among the two groups. Furthermore, we estimated the PFS and response rate based on the expression levels of MALAT1, HOTTIP and PVT1. The response rate of two groups showed a significant difference according to the expression levels of MALAT1, HOTTIP and PVT1. Based on the expression levels of MALAT1, HOTTIP and PVT1, the response rate of high expression of PVT1 and low expression of PVT1 was respectively 14.8% and 37.1% and 18.2% (high HOTTIP group) and 37.9% (low HOTTIP group), 10.7%(high MALAT1 group) and 41.1% (low MALAT1 group). The PFS of patients with high and low expression levels PVT1 was 2.6 months and 4.0 months (p<0.001), respectively. The PFS of patients with high and low expression levels of HOTTIP was 2.7 months and 4.1 months (p<0.001), respectively, and the PFS of patients with high and low expression levels of MALAT1 was 3.0 months and 3.7 months (P=0.026), respectively. The results suggest that MALAT1, HOTTIP and PVT1 as predictors to predict the efficacy of GEM based chemotherapy in first-line treatment of pancreatic cancer patients.

## INTRODUCTION

Pancreatic cancer is one of the most aggressive cancers with a high mortality rate in the world [1]. Despite the efforts of surgery, chemotherapy and radiation, the five-year survival rate is still less 5% and median survival time is about 4-6 months [2], Because of early metastasis and resistant to chemotherapy and radiation therapy [3]. Due to lack of specific early symptoms, most patients appear an advanced stage of disease at the time of diagnosis. For patients with advanced stage, chemotherapy plays an important role. Although the development of chemotherapy, gemcitabine-based therapy remains the standard first line treatment [4]. To date, carbohydrate antigen 19–9 (CA19-9) has certain role to predict the efficacy, but the specificity and sensitivity are very low [5]. Therefore, it is urgent to search for a sensitive biomarker to predict the efficacy of gemcitabine-based therapy.

Long non-coding RNAs (lncRNA) are a class of noncoding RNA with more than 200 nucleotides, but lack protein coding capacity [6, 7]. More and more investigations have demonstrated that lncRNAs exert an important role in proliferation, poptosis and migration. The expression levels of certain lncRNAs are associated with cancer recurrence, cancer progression, metastasis, and poor prognosis [8–10]. Some studies have reported that some lncRNAs such as H19, HOTAIR, HOTTIP, MALAT1,PVT1,ENST00000480739,Gas5,AF339813,LOC389641,AFAP1-AS1, BC008363, GAS,HMlincRNA717,HOTAIRM1,HULU [11–19] are most closely associated with PC. Therefore, lncRNAs might help to provide novel potential molecular markers for predicting the efficacy of gemcitabine-based.

Some studies shown that lncRNAs can be readily detected by qPCR from the body fluids, i.e. serum, plasma, gastric liquids, or urine [20]. In our study, we evaluated the levels of some lnc-RNAs in peripheral blood of patients with pancreatic cancer and analysed the correlation between lnc-RNAs, Progression-Free-Survival (PFS) and Response rate.

## RESULTS

### Patient characteristics

In our study, all patients were histologically diagnosed with pancreatic adenocarcinoma and were classified as locally advanced or advanced pancreatic cancer by CT. Total 62 patients received the GEM based chemotherapy as first-line treatment. According to the PFS, we divided into less than three months groups of PFS (29 patients) and greater than three months groups of PFS (33 patients). The two group patients have no significant difference in sex, age, phase and chemotherapy regimens. The characteristics of the two group patients are summarized in Table 1.

**Table 1:** Patients’ characteristics

Factor	PFS≤ 3 months groups	PFS>3 months groups	P
Median age (range)	56.5 (37–68)	59.0 (36–69)	
Gender			0.62
Male	18	14	
Female	15	15	
Performance status			0.40
0-1	17	18	
2	16	11	
First line regimen			0.38
Gemcitabine/Cisplatin	11	13	
Gemcitabine/Nab-paclitaxel	6	8	
Gemcitabine/Oxaliplatin	15	9	0.66
Clinical stage			
III	12	9	
IV	21	20	
Tumor location			0.60
Head	23	20	
Body	7	8	
Tail	3	1	
Differentiation			0.47
Well	19	15	
Moderate	8	5	
Poor	6	9	

### Response

In the two groups, the expression levels of three lnc-RNAs (MALAT1, HOTTIP, PVT1) had a significant difference (Table 2). Furthermore, we divided patients into high and low expression levels of lncRNAs groups based the MALAT1, HOTTIP, PVT1. The response rate of two groups showed a significant difference according to the expression levels of MALAT1, HOTTIP, PVT1. The Response rate of high expression of PVT1 and low expression of PVT1 was respectively 14.8% and 37.1% and had a significant difference (Table 3). Based on the expression level of HOTTIP, MALAT1, the Response rate was respectively 18.2%(high HOTTIP group) and 37.9% (low HOTTIP group) and 10.7%(high MALAT1 group) and 41.1% (low MALAT1 group) (Tables 4 and 5).

**Table 2:** The expression levels of lnc-RNAs according to the PFS

	Overexpression level		Downregulation level		P
	PFS<3months	PFS>3months	PFS<3months	PFS>3months	
H19	12	16	15	19	0.92
AFAP1-AS1	13	18	9	22	0.28
PVT1	19	8	10	25	<0.01
HOTAIR	16	19	14	13	0.63
HOTTIP	23	10	11	18	<0.01
MALAT1	20	8	13	21	<0.01
ENST00000480739	9	20	11	22	0.84
Gas5	18	16	15	13	0.96
AF339813	14	19	11	18	0.72
LOC389641	8	20	10	24	0.94
BC008363	10	22	13	17	0.32
HMlincRNA717	20	8	24	10	0.94
HOTAIRM1	15	19	12	16	0.92
HULU	13	18	15	16	0.61

**Table 3:** The difference of response according to the level of PVT1

Response	High *PVT1 (27)*	Low *PVT1 (35)*	P
CR	0	0	
PR	4 (14.8%)	13 (37.1%)	
SD	10 (37.0%)	15 (42.9%)	
Response rate	4 (14.8%)	13 (37.1%)	<0.001
Median PFS (95% CI)	2.6 (2.2-2.9) months	4.0 (3.5-4.5) months	<0.001

**Table 4:** The difference of response according to the level of HOTTIP

Response	High *HOTTIP (33)*	Low *HOTTIP (29)*	P
CR	0	0	
PR	6 (18.2%)	11(37.9%)	
SD	7 (21.2%)	13 (44.8%)	
Response rate	6 (18.2%)	11 (37.9%)	<0.001
Median PFS (95% CI)	2.7 (2.3-3.1) months	4.1 (3.6-4.7) months	<0.001

**Table 5:** The difference of response according to the level of MALAT1

Response	High *MALAT1 (28)*	Low *MALAT1 (34)*	P
CR	0	0	
PR	3 (10.7%)	14 (41.1%)	
SD	9 (32.1%)	16(47.1%)	
Response rate	3 (10.7%)	14 (41.1%)	0.007
Median PFS (95% CI)	3.0 (2.6-3.3) months	3.7 (3.17-4.26) months	0.026

**Figure 1:**
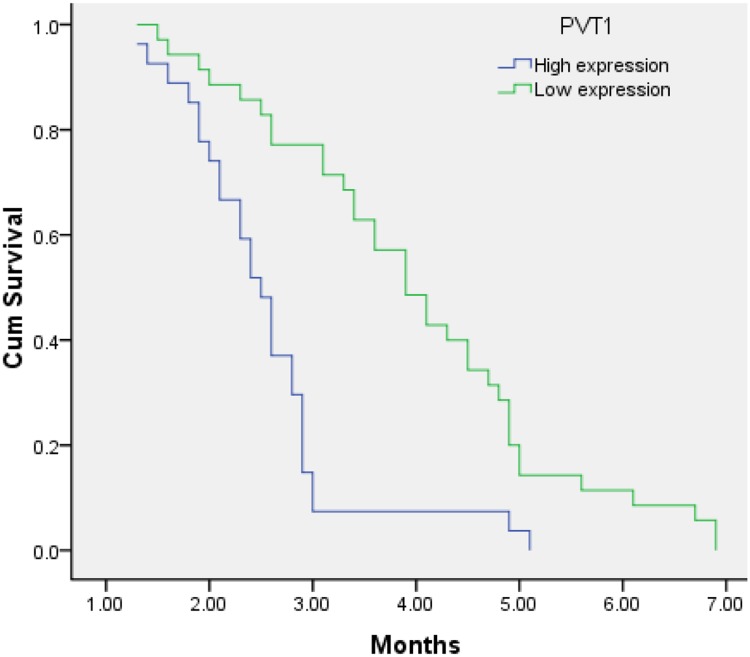
Kaplan–Meier analysis for PFS in pancreatic cancer patients with High expression level of PVT1 and low expression level of PVT1 (P < 0.001).

**Figure 2:**
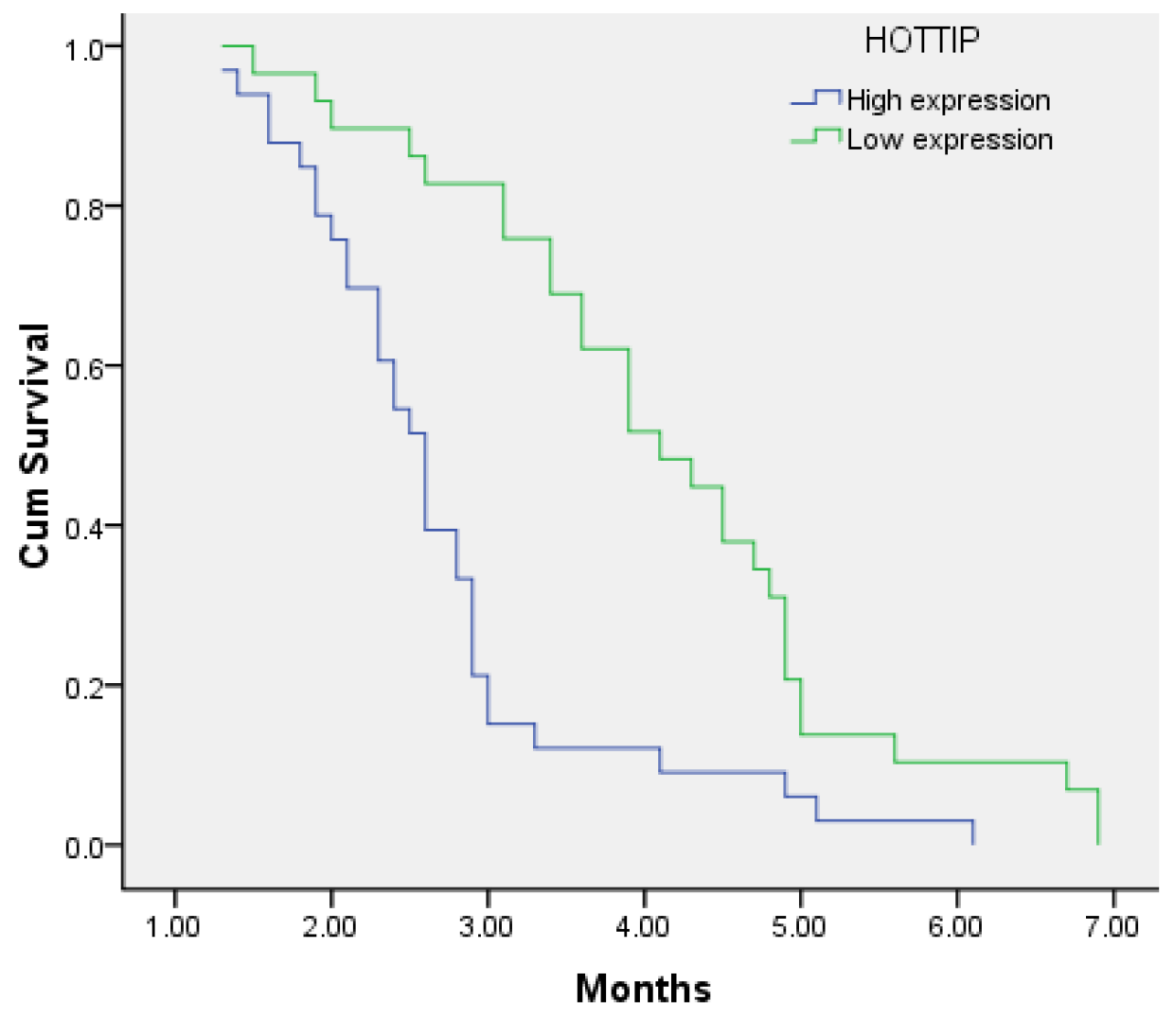
Kaplan–Meier analysis for PFS in pancreatic cancer patients with high expression level of HOTTIP and low expression level of HOTTIP (P < 0.001).

**Figure 3:**
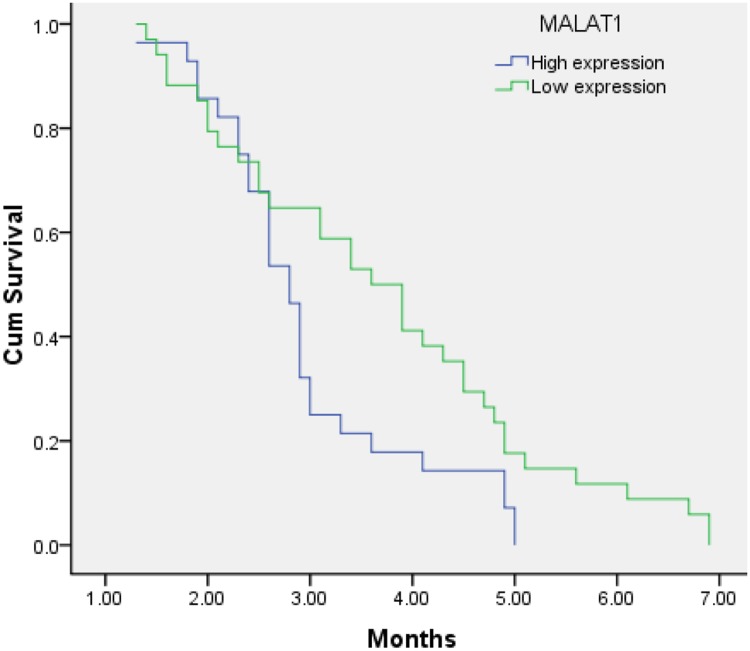
Kaplan–Meier analysis for PFS in pancreatic cancer patients with high expression level of MALTA1 and low expression level of MALTA1 (P < 0.026).

According to the expression levels of PVT1, MALAT1, HOTTIP, the PFS showed a significant difference. The PFS of patients with high and low expression levels PVT1 was 2.6 months (95% CI, 2.2-2.9) and 4.0 months (95% CI, 3.5-4.5) (p<0.001), respectively (Figure 1). The PFS of patients with high and low expression levels of HOTTIP was 2.7 months (95% CI, 2.3-3.1) and 4.1 months (95% CI, 3.6-4.7) (p<0.001), respectively, and the PFS of patients with high and low expression levels of MALAT1 was 3.0 months (95% CI, 2.6-3.3) and 3.7 months (95% CI, 3.2–4.3) (P=0.026), respectively (Figures 2 and 3).

## DISCUSSION

At present, pancreatic cancer remains a highly malignant tumor and the prognosis is extremely poor. For many years, new drugs for cancer treatment have been appeared consecutively, but Gemcitabine-based treatment remained the first-line chemotherapy regimen for patients with advanced pancreatic cancer [21]. Although, the level of serum CA19-9 may help to predict the prognosis partly, the situation is unsatisfying the need of individualized therapy [22, 23]. Therefore, we have focused on searching reliable biomarkers to predict the therapeutic efficacy and avoid some ineffective patients to receive aggressive therapies.

LncRNAs were found to be more stable in that case situation of multiple freeze-thaw cycles, strong acid and base. Some studies have demonstrated that dysregulated expression of lncRNAs may independently predict patient outcome [24, 25]. Li et al. found that the expression of HOTTIP was up-regulated in pancreatic cancer tissues and demonstrated that HOTTIP was over-expressed in cancer tissues compared with non-tumoral tissues and elevated the ability of pancreatic cell proliferation and invasion. Targeted silencing of HOTTIP may enhance the antitumor effects of gemcitabine [12]. Furthermore, expression of HOTTIP has been identified as a negative prognostic factor in hepatocellular carcinoma patients [26]. Another study shows that HOTTIP increases pancreatic cancer cell proliferation, survival, and migration through HoxA family genes other than HoxA13 [27].

One study has shown that PVT1 gene could regulate Gemcitabine sensitivity in human pancreatic cell line. The sensitivity of Gemcitabine was changed according to the situation of antisense orientation and sense orientation of full length PVT1 cDNA.

Overexpression of PVT1 decreased gemcitabine sensitivity, while functional inactivation of the PVT1 enhanced Gemcitabine sensitivity [28]. Mechanism study demonstrated that the upregulated PVT1 could induce cell cycle genes and activation of the TGFβ1 signaling pathway and PVT1 binds to EZH2, recruits EZH2 to the miR-200b promoter, which increases histone H3K27 trimethylation level on the miR-200b promoter, and inhibits miR-200b expression [29, 30]. Knockdown of MALAT1 expression inhibited PC cell proliferation, migration, and invasion *in vitro* by inducing G2/M cell cycle arrest, suppressing EMT and decreasing cancer stem-like properties [13, 31]. In our study, the high and low expression level of lnc-RNAs had a different PFS and response rate. Above studies may also provide a certain confirmation.

In conclusion, our study suggested that PVT1, HOTTIP, MALAT1 was associated with the efficacy of first-line treatment with GEM based chemotherapy in pancreatic cancer. They may be novel non-invasive biomarkers for predicting the efficacy. The limitation of our study was that the sample size was small and a larger sample size is needed to confirm the results in the future. In-depth analysis of their biological functions is needed.

## MATERIALS AND METHODS

### Patients

The patients who take part in this study had histologically proven advanced pancreatic cancer. All patients received GEM based chemotherapy in first-line treatment between February 2014 and January 2016 at Shandong Tumor Hospital. The patient selection criteria were: age, 18–75 years old; ECOG performance status (PS) ≤2; adequate hematological, hepatic function adrenal functions (white blood cell count ≥4.0×10^9^/l; neutrophil count ≥1.5×10^9^/l; platelet count ≥100×10^9^/l and hemoglobin≥10 g/dl; ALT and AST ≤ 2.5 ×upper limits of normal; total bilirubin ≤1.5×upper limits of normal; creatinine clearance ≥ 60 ml/min or creatinine ≤ upper limits of normal); and life expectancy greater than 3 months. All the patients had at least one measurable lesions by CT scan or MRI. Exclusion criteria were massive pleural effusion or ascites, active concomitant malignancy, brain metastasis, pregnant or lactating if patients were women. All the patients were informed, and the trial was authorized by the ethics committee of Shandong tumor hospital.

### Detection serum levels of lncRNAs

Blood samples were collected before chemocherapy and then centrifuged at 3000 × g for 10 min, and stored in RNase- and DNase-free tubes at –80 °C before use. Serum RNA isolation was performed using a Blood Total RNA Isolation Kit (RP4001, BioTeke, Beijing, China) according to manufacturer's protocol. RT and qPCR kits were used to evaluate the expression levels of the selected lncRNAs in the samples as described previously [32]. β-actin was used as control. The relative expression levels of each lncRNA was calculated using the 2^–ΔΔCt^ method and was normalized to that of β-actin.

### Treatment and evaluation

All patients received GEM based chemotherapy, including gemcitabine plus cisplatin, gemcitabine plus Nab-paclitaxel or gemcitabine plus Oxaliplatin. The dosages were described previously [33, 34]. Treatment continued until a maximum of 6 cycles, progressive disease, unacceptable toxicity occurred, or withdrawal of patient consent. Before treatment, all patients were collected blood to test the level of serum lnc-RNAs and carbohydrate antigen (CA) 19-9(CA19-9). Tumor responses were evaluated every two cycles of treatment via computed tomography (CT) according to the Response Evaluation Criteria in Solid Tumors (RECIST, ver. 1.1).

### Statistical analysis

All statistical analyses were performed using the SPSS 17.0 software package (SPSS Inc., Chicago, IL, USA). The correlations were analyzed using the Student's t-test, the Chi-square test and analysis of variance (ANOVA). Survival curves were analyzed according to the Kaplan-Meier method. All statistical tests were two tailed and results with P = 0.05 were defined as statistically significant.

## Abbreviations

GEM: gemcitabine
CA19-9: carbohydrate antigen
lncRNA: long non-coding RNAs
PFS: progression-free-survival
PS: performance status
CT: computed tomography
RECIST: response evaluation criteria in solid tumors
